# Novel *ABCD1* variant causes phenotype of adrenomyeloneuropathy with cerebral involvement in Ukrainian siblings: first adult hematopoietic stem cell transplantation for ALD in Ukraine: a case report

**DOI:** 10.1186/s13256-023-04321-1

**Published:** 2024-01-21

**Authors:** Khrystyna Shchubelka, Olga Herasymenko, Andrii Budzyn, Oleksandr Lysytsia, Anastasiia Rusyn, Olga Oleksyk, Svitlana Tynta, Taras Oleksyk

**Affiliations:** 1 Department of Biological Sciences, Oakland University, 118 Library Drive, Rochester, MI 48309 USA; 2 Department of Biology, State University “Uzhhorod National University”, Voloshyna Street, 32, Uzhhorod, 88000 Ukraine; 3 Regional Centre of Neurosurgery and Neurology, Uzhhorod, 88000 Transcarpathian Region Ukraine; 4 Bone Marrow Transplantation and Immunotherapy Department, NSCH “Okhmatdyt”, Kiev, Ukraine; 5 Department of Medicine, State University “Uzhhorod National University”, Narodna Square, 1, Uzhhorod, 88000 Ukraine; 6 Zakarpattia Regional Clinical Hospital, Kapushanska 22, Uzhhorod, 88000 Ukraine

**Keywords:** Adrenoleukodystrophy, Adrenomyeloneuropathy, ABCD1 gene, Novel variant, Hematopoietic stem cell transplant

## Abstract

**Background:**

This article presents a case study of two white male siblings of 24 and 31 years of age of self-reported Ukrainian ethnicity diagnosed with adrenomyeloneuropathy (AMN) associated with a novel splice site mutation in the *ABCD1* gene. AMN represents a form of X-linked adrenoleukodystrophy (X-ALD) characterized by demyelination of the spinal cord and peripheral nerves. The case also presents the first adult haematopoietic stem cell transplant (HSCT) for adrenomyeloneuropathy in Ukraine. The rarity of this mutation and its cerebral involvement and the treatment make this case noteworthy and underscore the significance of reporting it to contribute to the existing medical knowledge.

**Case presentation:**

The patients of 24 and 31 years initially exhibited progressive gait disturbance, lower extremity pain, and urinary incontinence, with the older sibling experiencing more advanced symptoms of speech, hearing, and vision disturbances. A comprehensive genetic analysis identified an unreported splice site mutation in exon 3 of the *ABCD1* gene, leading to the manifestation of AMN. The inheritance pattern was consistent with X-linked recessive transmission. The article also outlines the clinical features, magnetic resonance imaging (MRI), and nerve conduction study (NCS) findings. Moreover, it discusses the genetic profile of the affected individuals and female carriers within the family. The younger sibling underwent HSCT, which was complicated by mediastinal lymph node and lung tuberculosis, adding to the complexity of managing adult ALD patients.

**Conclusions:**

This report emphasizes the importance of genetic testing in diagnosing and comprehending the underlying mechanisms of rare genetic disorders, such as AMN with cerebral involvement. The identification of a novel splice site mutation expands our understanding of the genetic landscape of this condition. Additionally, the challenges and complications encountered during the hematopoietic stem cell transplant procedure underscore the need for cautious consideration and personalized approaches in adult ALD patients.

## Introduction

X-linked adrenoleukodystrophy (X-ALD) is a relatively common peroxisomal genetic disorder that affects approximately 1 in 15,000–17,000 individuals worldwide. The disease is caused by various mutations in the *ABCD1* (Xq28) gene, which codes for adrenoleukodystrophy protein (ALDP) [1]. This protein acts as a transmembrane transporter of very long-chain fatty acids (VLCFAs) into peroxisomes for subsequent β-oxidation. Elevated levels of specific acids, such as tetracosanoic acid (C24:0) and hexacosanoic acid (C26:0), as well as their ratios to docosanoic acid (C22:0), serve as early diagnostic markers for ALD [2]. While the exact pathophysiological mechanisms are not fully understood, the accumulation of unprocessed VLCFAs disrupts glial homeostasis and leads to myelin damage throughout the nervous system [3]. ALD exhibits highly variable expressivity and is characterized by four primary clinical forms: childhood cerebral ALD, adult cerebral ALD, adrenomyeloneuropathy (AMN), and isolated Addison's disease (adrenal insufficiency only) [4]. Female carriers may also show neuro- and myopathy symptoms typically in lower limbs [5].

AMN affects 40 to 60% of ALD patients and involves demyelination of the spinal cord and peripheral nerves, presenting as spastic paraplegia with bladder dysfunction and Addison's disease. Adrenal insufficiency typically precedes neuropathy by several decades and begins in early childhood [6].

In this report, we present a novel splice site mutation in the *ABCD1* gene that resulted in a highly similar AMN phenotype with cerebral involvement (cALD) in adulthood in two brothers. Given the considerable variability in ALD presentations, this case represents a rare occurrence of nearly identical phenotypes presented nearly at the same age. Additionally, we describe the magnetic resonance imaging (MRI) and nerve conduction study (NCS) test findings in the affected males.

## Case presentation

### Clinical features

A 24-year-old white male (proband) presented to the Neurology department of Zakarpattia Regional Hospital, Uzhhorod, Ukraine with progressive gait disturbance, lower extremities spasticity and pain (5/10 points, where a score of 0 means no pain, and 10 means the worst pain one has ever experienced), and urinary incontinence. The leg pain had been slowly progressing over 6 months before the visit, the patient described it as resembling muscle pain during flu infection. He had a history of primary adrenal insufficiency since the age of 4. Proband’s older brother (31 years old) also experienced primary adrenal insufficiency starting at the age of 8. Both are of Ukrainian ethnicity from Ukraine. Genetic testing and VLCFA analysis were not performed due to unavailability in Ukraine at that time. Both patients have been receiving hydrocortisone (35 mg/d) and fludrocortisone (0.05 mg/d) replacement therapy following their diagnoses. The older brother developed a methamphetamine addiction at the age of 25, and at 28, he exhibited gait and speech disturbances that were initially attributed to drug abuse. At the age of 23, the proband experienced lower extremity pain, leading to the suspicion of AMN and establishing a connection between his symptoms and those of his brother. Both patients had also thin scalp hair.

### Genetic testing results

**Fig. 1:**
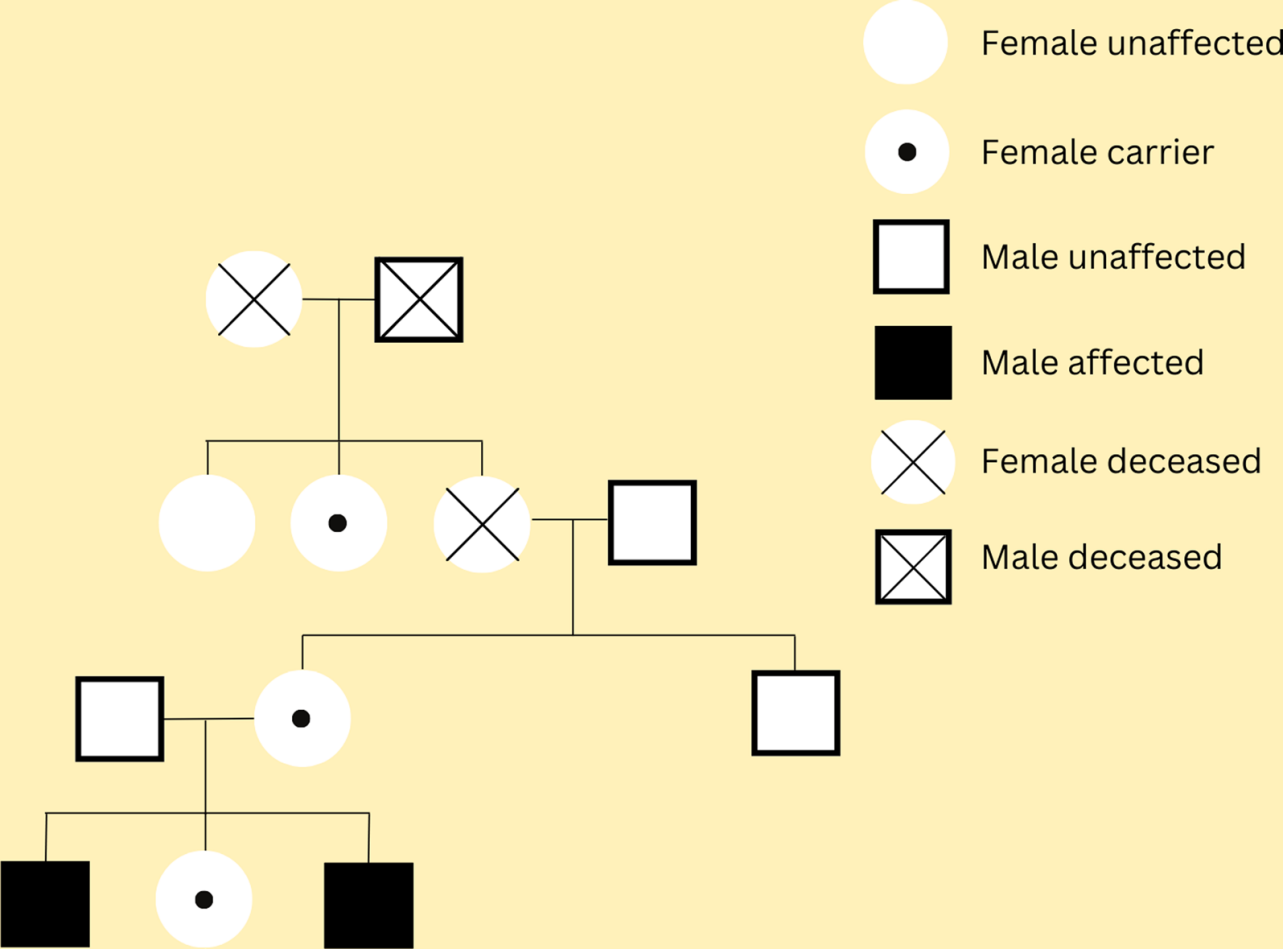
Pedigree of the extended family with the novel c.1224G > A, Glu408 = mutation in *ABCD1* gene

Next generation sequencing (NGS) sequencing of the *ABCD1* gene in the proband unveiled a previously unreported splice site mutation in exon 3 (c.1224G > A, Glu408 =), leading to the disruption of the consensus splice site. The same mutation was identified in the older brother, with hemizygous inheritance, while the mother and sister carried the mutation in a heterozygous state. Comprehensive testing was conducted on all maternal extended family members, revealing an additional carrier of the mutation (grandmother's sister). The family pedigree is depicted in Fig. 1.

There is no information on this variant in the population databases (no entry in Gnomad), the frequency in the Ukrainian sample of 247 healthy individuals is 0.

Clinvar database contains the entry for this variant from Invitae without the supporting evidence of affected status (variation ID: 1455540).

### Hormonal and VLCFA profile in the family

VLCFA assay was positive and revealed significantly elevated C26:0 (hexacosanoic acid) in both brothers and slightly elevated levels in carrier sister (Table 1).

**Table 1:** Hormonal and VLCFA profile of the family

Marker	Proband	Proband’s brother	Proband’s Sister carrier	Normal values
ACTH	1250	254	NA	7–69 pg/ml
Cortisol (serum)	3.1	4.5	NA	4.3–22.4 μg/dl
C26:0	1.22	1.34	0.69	0.10–0.60 mg/l
C24:0	29.80	42.60	28.30	8.50–35.70 mg/l
C22:0	17.10	40.5	23.10	10.50–51.00 mg/l
C26:0/C22:0	0.07	0.4	0.03	< 0.04
C24:0/C22:0	1.74	1.34	1.23	< 1.16

Carrier mother was not tested

*ACTH* adrenocorticotropic hormone; *C26:0* Hexacosanoic acid; *C24:0* Tetracosanoic acid; *C22:0* Docosanoic acid; *C26:0/C22:0* ratio of Hexacosanoic acid to Docosanoic acid; *C24:0/C22:0* ratio Tetracosanoic acid to Docosanoic acid

### MRI findings

**Fig. 2:**
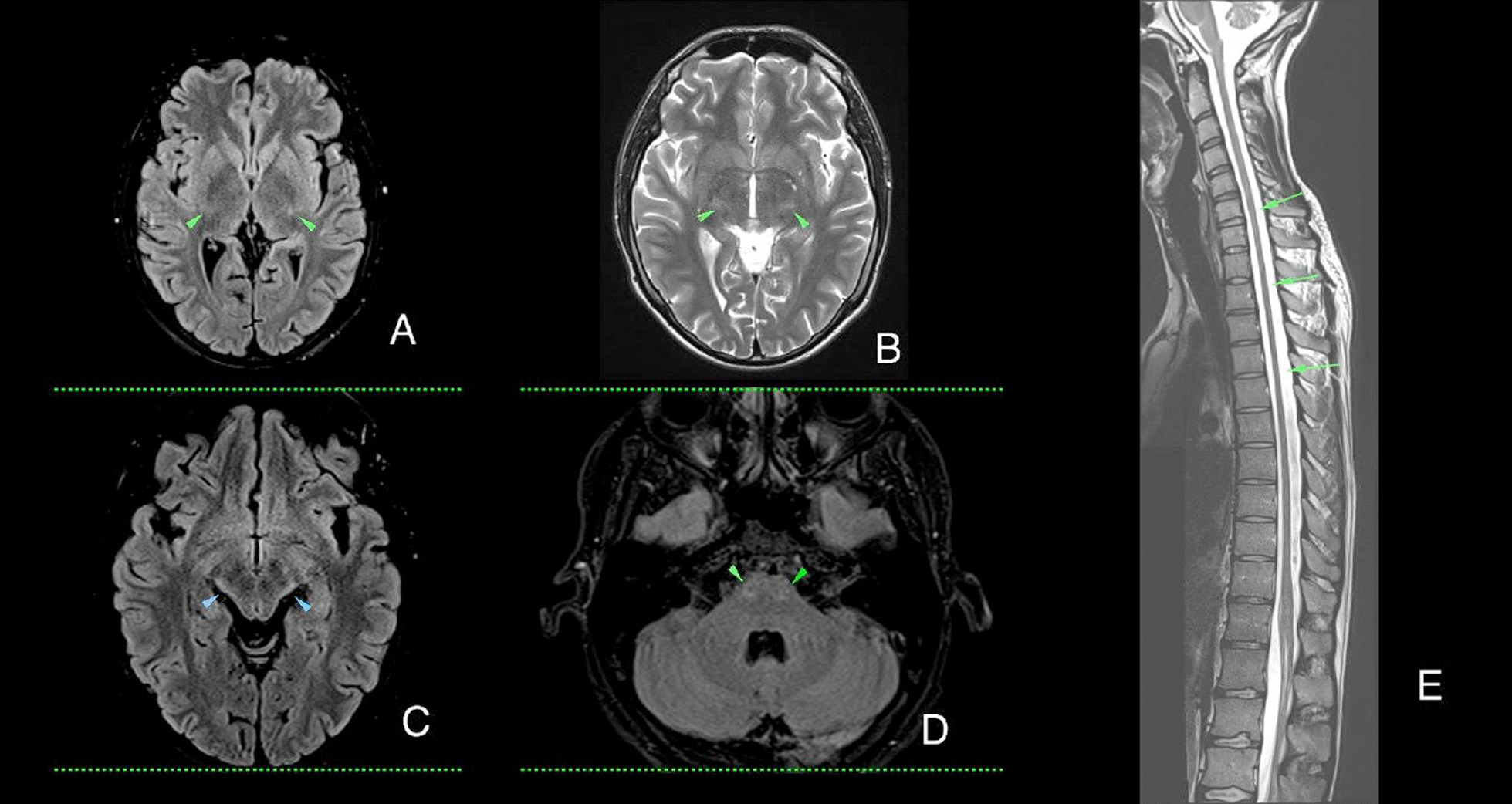
Pre-HCT MRI, 24 y o proband, T2, Flair hyperintensities of cortico-spinal tracts at various levels (green arrowheads **A**, **B**, **D**); symmetrical bilateral signal changes at the level of medial and lateral geniculate bodies (blue arrowheads, **C**); diffuse spinal cord atrophy (green arrowhead, **E**)

The severity of ALD white matter lesions is rated by MRI imaging-based Loes score (rages from 0 to 34). Usually the score over 7 is an cut-off for HSCT consideration [7]. The proband (24 years o) exhibited symmetric white matter hyperintensities in the periventricular parietooccipital white matter, (non specific), in the projection of medial and lateral geniculate bodies, and also in the corticospinal tract at various levels including the posterior limb of the internal capsule, midbrain, pons, and medulla (Fig. 2). The Loes score [7] for the 24-year-old proband was 4, while the 31-year-old brother scored 11.

### NCS findings

Lower extremities NCS in the proband found non-inflammatory axonal damage, especially left deep peroneal nerve at the level of plantar joint. All available family members underwent electrodiagnostic study. Each NCS included sensory and motor studies, and needle electromyography (EMG) assessed spontaneous activity.

#### Results

24 years o (Proband, affected)

- NCS: distal latencies, amplitudes, and conduction velocities were normal. High amplitude and size of F-waves—1371 uV
- EMG: segmental positive sharp waves detected and motor unit potential configuration (MUAPs) were short in duration (7.74 ms) and had low amplitude (199–2438 uV) with early recruitment pattern.

31 years o (Proband’s brother, affected)

- NCS included sensory and motor studies (normal distal latencies and amplitudes, slow conduction velocities 25.5 m/s). High amplitude and size of F-waves-1707 uV, long latencies—63.9 ms
- EMG: segmental fibrillations detected and motor unit potential configuration (MUAPs) we normal in duration 10 ms and low/high amplitude 205–3935 uV with early recruitment pattern.

56 years o (Proband’s mother, carrier)

- NCS: normal distal latencies and amplitudes, slow conduction velocities (43.7 m/s). Normal amplitude and size of F-waves—830 uV
- EMG: segmental fibrillations detected and motor unit potential configuration (MUAPs) were normal in duration (17 ms) and had low/high amplitude 290–520 uV with early recruitment pattern.

26 years o (Proband’s sister, carrier)

- NCS: normal distal latencies and amplitudes, slow conduction velocities (43.1 m/s). Normal amplitude and size of F-waves—955 uV
- EMG: No spontaneous activity detected, motor unit potential configuration (MUAPs) (normal in duration (14–18 ms) and normal/high amplitude 245–1670 uV with early recruitment pattern. The electrodiagnostic study results in the family are summarized in the Table 2.

**Table 2:** Electrodiagnostic study results

Nerve conduction studies (NCS)					
Case	1	2	3	4	
Gender and status	Male (proband) affected	Male (proband’s brother) affected	Female (proband’s mother) carrier	Female (proband’s sister) carrier	
Age	23	31	56	26	
Motor					
Distal latencies	N	N	N	N	2.5–3 ms
Amplitudes	N	N	N	Decreased	3–8 mV
Conduction velocities	N	Slow	Slow	Slow	40–60 m/s
Sensory					
Distal latencies	N	N	N	N	2 ms
Amplitudes	N	N	N	Decreased	5–15 µV
Conduction velocities	N	Slow	Slow	Slow	50 m/s
F-waves					
Amplitudes	High	High	N	N	200–1000 µV
Size	Huge	Huge	N	N	˃1000 µV
Latencies	N	Long	Long	Long	40–50 ms
Needle EMG (EMG)					
Spontaneous activity	Segmental positive sharp waves	Segmental fibrillations	Segmental fibrillations	–	None
Motor unit potential configuration (MUAPs)					
Duration	Short	Normal	Long	Long	9–11 ms
Amplitude	High—upper limbs Low—lower limbs	High—upper limbs Low—lower limbs	Low	High-upper limbs Normal—lower limbs	200–900 µV
recruitment pattern	Early	Early	Early	Early	1200–2500 µV
Neurological status					
Normal reflexes	High	High	High	High	Norm rate
Pathological reflexes	+	+	−	−	None
Conclusion	Motoneuron activity—*neurogenic* Secondary myopathy	Motoneuron activity—*neurogenic* Secondary myopathy Sensory polyneuropathy, with demyelination	Secondary myopathy Distal sensory polyneuropathy	Motoneuron activity—*neurogenic* Secondary myopathy Sensory polyneuropathy	

Based on all the test and clinical examination the diagnosis of AMN with cerebral involvement was established.

### Results of hematopoietic stem cell transplant

#### Conditioning and immune reconstitution

Given the nature of the disease, the progression of adrenomyelopolyneuropathy with cerebral involvement, the patient underwent allogeneic hematopoietic stem cell transplantation (HSCT) from match unrelated donor (10/10 MUD). The source of the graft was peripheral blood stem cells (PBCS). A comprehensive pre-transplantation examination of the patient was carried out before HSCT. Before HSCT was conducted laboratory-instrumental pre-transplantation examination of the patient. Absolute contraindications to the transplantation procedure were not found.

A reduced-intensity conditioning (RIC) was performed according to the following scheme: Treosulfan 36 g/m^2^, Fludarabine 150 mg/m^2^, Thiotepa 10 mg/kg. Due to the high risk of graft-versus-host disease (GVHD) associated with allo-HSCT from MUD, antithymocyte globulin [Thymoglobulin (anti-thymocyte globulin rabbit 7.5 mg/kg)] was added.

Cyclosporin A (CsA) and Methotrexate (Mtx) were chosen as classical prophylaxis of GVHD. CsA at a dose of 3 mg/kg/day, starting on day-1, while maintaining serum levels between 100 and 150 ng/ml. From day + 120, taking into account the absence of manifestations of GVHD, infectious complications after HSCT in the form of tuberculosis of the mediastinal lymph node, a decision was made to gradually withdraw CsA within two months.

All three engrafts were achieved at the estimated time, without delays. Specific complications after HSCT of immune genesis (engraftment syndrome, GVHD) were not observed. The dynamics of immune cell reconstitution [8] after HSCT is shown in the Table 3.

**Table 3:** Immune reconstitution dynamics (days after HSCT)

Subpopulations of lymphocytes			
	DAY + 26	DAY + 73	Normal range
Total count	3220	3340	4000–9000
CD45/C19	0	0	60–680
CD45/CD3	450	740	440–2740
CD45/CD3/CD4	110	70	270–1980
CD45/CD3/CD8	190	630	130–1330
NK (CD16/CD56	130	40	70–580
CD4/CD8 coefficient	0,6	0,12	1.1–2.5

**Fig. 3:**
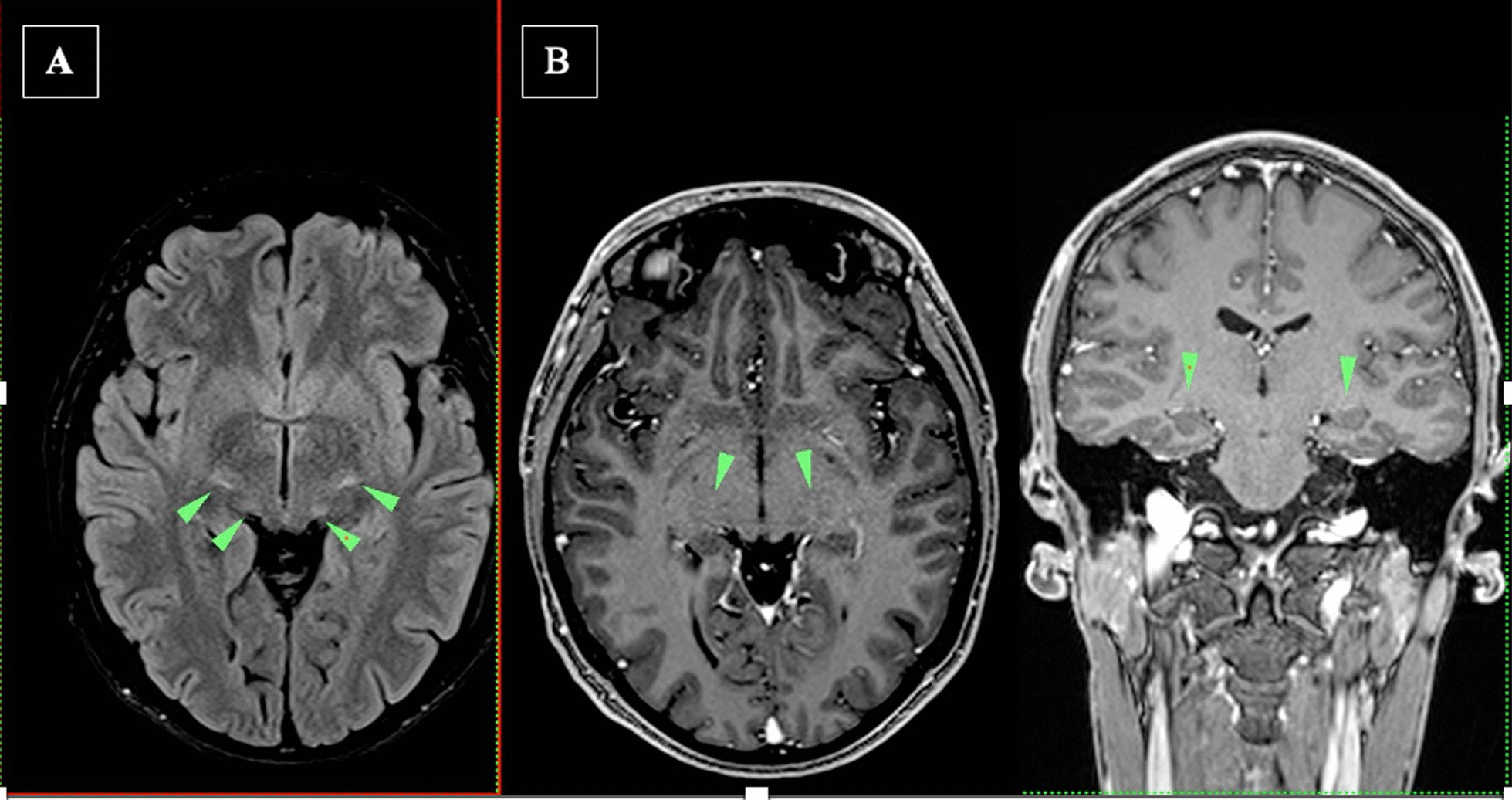
MRI+ 90 day post HCT. No changes comparatively to pre HCT findings (Flair hyperintensities of cortico-spinal tracts at various levels and geniculate bodies (green arrowheads **A**, **B**)

MRI on the day 90 post HSCT reveled no changes comparatively to the pre HSCT MRI results (Fig. 3).

The 31-year-old proband’s brother was not considered for HSCT due to the advanced symptoms of adrenoleukodystrophy (ALD) and the corresponding MRI findings, including a high Loes score of 11.

## HSCT complications in the proband

From Day + 51, the patient had persistent fever. Active differential diagnosis between fever of an infectious or immune nature was carried out. On the background of combined systemic antibacterial therapy, which was prescribed taking into account the patient's microbial culture no positive effect was observed. The fever continued to persist. Laboratory markers of the systemic inflammatory response remained at a consistently high level, however, their level did not indicate complex septic processes in the body. In the process of additional laboratory and instrumental examination, fungal and viral infections were excluded.

CT diagnostics revealed solid nodules in the lungs and an enlarged paratracheal lymph node with signs of destruction, necessitating differentiation between fungal, tuberculosis, and Mycobacterium avium complex (MAC) infection. In the conditions of the surgical department, a video thoracoscopy was performed, as well as a resection followed by a biopsy of the removed lymph node. Further histology of excised lymph node supported the diagnosis of active tuberculous lymphadenitis and newly diagnosed pulmonary tuberculosis (S 6, lower lobe of the right lung). Joint curation was mostly carried out under the supervision of a hematologist and a phthisiologist. The patient was initiated on therapy with a combination of isoniazid, rifampicin, ethambutol, and pyrazinamide (HREZ) under the supervision of a pulmonologist. Later, in response to ongoing immunosuppressive therapy and the progression of tuberculosis infection, the systemic antibiotic therapy was adjusted to include the synergistic anti-tubercular activity of moxifloxacin and linezolid.

However, febrile episodes and high inflammatory markers persisted during therapy, requiring continuous clinical and laboratory monitoring. Given the immunocompromised result of IST, there was constant control of other bacterial and viral infections, potential immune complications and IRIS syndrome. Approximately after 3 months of treatment, the patient began to observe a gradual decrease in the markers of the systemic inflammatory response, “normalization” of the temperature curve. Unfortunately, the treatment of this complication is long-term, difficult to control, and requires careful and intensive monitoring with the involvement of numerous specialists. The overall course of tuberculosis treatment was challenging, with nonspecific CT findings and ongoing systemic inflammation, highlighting the complexity of the patient’s condition.

## Discussion

This case report describes the clinical features, genetic findings, and treatment outcomes of two siblings with adrenomyeloneuropathy associated with a novel splice site mutation in the *ABCD1* gene. The patients presented with progressive gait disturbance, lower extremity pain, and urinary incontinence, which are characteristic symptoms of AMN [1].

The genetic analysis identified a previously unreported splice site mutation in exon 3 of the *ABCD1* gene in both affected siblings. This mutation led to the disruption of the consensus splice site and resulted in the manifestation of AMN. The inheritance pattern was consistent with X-linked recessive inheritance, as the mutation was hemizygous in the affected brother and heterozygous in the carrier mother and sister. Additionally, an extended family analysis revealed another carrier of the mutation (grandmother’s sister).

The MRI findings in the proband showed symmetric white matter hyperintensities in the corticospinal tract at various levels, indicating the involvement of the central nervous system. The Loes score, which is used to assess the severity of demyelination in AMN, was higher in the older brother compared to the proband, suggesting a more advanced disease stage in the former.

While mutation type typically fails to accurately predict the phenotype of ALD in the majority of cases [9], our study provides a unique perspective as the two brothers in our case displayed the same phenotype, albeit at different ages, emphasizing the significance of age-related variations in disease progression.

Electrodiagnostic studies, including nerve conduction studies (NCS) and needle electromyography (EMG), were conducted to evaluate the peripheral nerve involvement in the patients. The proband exhibited non-inflammatory axonal damage in the lower extremities, while the older brother showed slow conduction velocities and motor unit potential abnormalities. These findings were consistent with a neurogenic secondary myopathy and motor neuron dysfunction. The carrier mother and sister had normal NCS and EMG results, indicating that they did not show signs of peripheral nerve involvement.

The proband underwent HSCT from an HLA-matched unrelated donor. The conditioning regimen included cyclosporin A and methotrexate for GVHD prophylaxis. The patient achieved complete donor cell engraftment on day 73, indicating successful hematopoietic reconstitution. However, the transplantation procedure was complicated by the development of mediastinal lymph node tuberculosis, which required intensive anti-TB treatment.

Comparing this case report to other studies in the literature, the clinical features and genetic findings of AMN in these siblings are consistent with previously reported cases [2]. The identification of a novel splice site mutation expands the spectrum of *ABCD1* gene mutations associated with AMN and shows that it can cause very similar phenotype in one family.

The electrodiagnostic studies provided insights into the peripheral nerve involvement in AMN. The proband showed evidence of non-inflammatory axonal damage, while the older brother exhibited slow conduction velocities and motor unit potential abnormalities. These findings align with the neurogenic secondary myopathy and motor neuron dysfunction commonly observed in AMN. Both carrier mother and sister exhibited early signs of secondary myopathy and distal neuropathy. As commonly seen in carriers, proband’s grandmother who is deceased and was a presumable carrier had severe gait disturbance and leg pain progressing after the age of 65, misdiagnosed as arthritis.

The early successful results of hematopoietic stem cell transplantation in the proband highlights that HSCT is still the possibility in adulthood.

The development of mediastinal lymph node tuberculosis as a complication of the transplantation procedure emphasizes the importance of pre-HSCT detection of latent tuberculosis [10], especially in adults, and appropriate management of potential reactivation (prophylactic measures) in high-risk individuals specifically with adrenal insufficiency. Implementing pre-transplant routine screening for tuberculosis, especially in regions with a high prevalence of the disease, could help identify latent infections and allow for targeted prophylactic measures.

In fact, this issue is debatable and controversial. As of today, there is no single clear position among the medical community regarding the prevention of tuberculosis infection and pre-transplantation screening [4]. This includes countries where tuberculosis is a widespread and frequent disease. In most cases, recipients have reduced immunity to HSCT, as well as reduced T-cell immunity due to IST after HSCT. That is why the tuberculin skin test and/or QuantiFERON-TB gold (QFT-GIT), which are common today, have low effectiveness in detecting the latent form of tuberculosis. There is no consensus on the primary prevention of latent tuberculosis, and further research is needed on this issue as well [10].

Patients from the so-called high-risk group can be potential candidates for the prevention of reactivation of the latent form of tuberculosis infection. Preventive therapy, in particular with the drug isoniazid, may be indicated in patients with a previous history of active tuberculosis, contact with people with active, open tuberculosis, and patients with positive tuberculin skin test and /or interferon-gamma release assays (IGRA) [10].

Patient assessed his subjective physical condition on the day + 203 as pre-HSCT.

## Conclusions

In conclusion, this case report contributes to the understanding of AMN by describing a novel splice site mutation in the *ABCD1* gene and providing insights into the clinical features, genetic findings, and treatment outcomes. The findings are consistent with previous studies and highlight the complex nature of AMN and the challenges associated with its diagnosis and management. The case shows a strong potential for HSCT success in adulthood.

Unfortunately, data on the use of HSCT in AMN with cerebral involvement are extremely limited. This method can be the only radical method of slowing down the progression of irreversible changes in the central and peripheral nervous system. Unfortunately, as is known, the functions of the adrenal glands will be permanently damaged and lifelong hormone replacement therapy is required.

Prenatal diagnosis, newborn screening, genetic testing are the cornerstones of timely disease diagnosis.

## Data Availability

The data are available from the corresponding author on reasonable request.
